# Spirituality and Continued Abstinence Among Methamphetamine Users in a 12‐Step Program in Japan: Data From a Cohort Study of Residential Substance Use Treatment

**DOI:** 10.1002/npr2.70032

**Published:** 2025-07-10

**Authors:** Takuma Ofuchi, Takuya Shimane, Toshihiko Matsumoto

**Affiliations:** ^1^ Department of Drug Dependence Research National Center of Neurology and Psychiatry, National Institute of Mental Health Tokyo Japan; ^2^ Herefordshire and Worcestershire Health and Care NHS Trust Hereford UK

**Keywords:** 12‐step program, abstinence, drug use disorder, methamphetamine, spirituality

## Abstract

**Aim:**

This study investigated the relationship between spirituality and long‐term methamphetamine abstinence among the participants of a 12‐step‐based program in Japan.

**Methods:**

The participants were 120 individuals with methamphetamine use disorder who stayed in drug addiction rehabilitation centers in 2016. They were followed up with eight surveys conducted approximately every 6 months over 5 years. The survey data were analyzed using binary logistic regression, where the dependent variable was methamphetamine abstinence for 6 years. The explanatory variables were age, gender, scores on the Drug Abuse Screening Test‐20 (DAST‐20), and the Spiritual Rating Scale A (SRS‐A), changes in the frequency of group meeting attendance, group involvement, and treatment in a hospital.

**Results:**

Higher spirituality was significantly associated with long‐term methamphetamine abstinence (adjusted odds ratio [AOR] = 1.05, 95% confidence interval [CI] = 1.00–1.09). Program involvement was also significantly associated with abstinence (AOR = 2.28, 95% CI = 1.37–3.79), whereas the interaction between SRS‐A and DAST‐20 scores on abstinence was not significant (AOR = 1.00, 95% CI = 0.99–1.00).

**Conclusion:**

The level of spirituality may be positively related to methamphetamine abstinence rates among Japanese participants in a 12‐step‐based program, regardless of the severity of substance use disorder. The study results indicate the importance of spirituality for continued abstinence from methamphetamine, even among those who have a different understanding of spirituality from that in Western countries.

## Abstract 2

A cohort study of residential substance use treatment revealed the relationship between spirituality and methamphetamine abstinence among Japanese. The higher spirituality was significantly related to longer methamphetamine abstinence.

## Graphical abstract

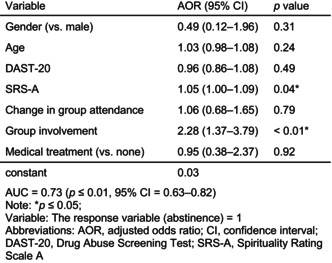

## 1 Introduction

Spirituality/religion (S/R) plays a role in protecting people from substance use and motivating recovery from substance use disorder. Spirituality refers to the basic human trait of seeking profundity, meaning, and well‐being through relationships with the universe, others, and oneself [1]. It adds a sense of meaning and purpose to life [2] and involves a sense of personal identity and transcendence that pushes individuals beyond the everyday problems of life [3]. According to Canda and Furman [1], a religion is an institutionalized set of values, beliefs, and practices geared toward spiritual issues that are passed down through a community over time. Although there are some similarities and differences between the two terms, they are often used interchangeably because of their similar roles in recovery from addiction. For example, strong religious or spiritual convictions are correlated with a lower likelihood of consuming alcohol as well as lower alcohol consumption [3, 4]. In addition, S/R may offer protection against substance use disorder [5, 6]. Some believe that alcoholism develops as a “spiritual” illness that can be healed by a “spiritual cure.” [7, 8, 9, 10, 11]

Thus, S/R is frequently mentioned in the recovery process of substance use disorder, especially self‐help groups such as Alcoholics Anonymous (AA), which is one of the most popular programs in the US for treating alcoholism. In AA, spiritual growth is a crucial part of recovery and a major part of the 12‐step program [12, 13, 14, 15]. The goal of the 12‐step program is to help people make the internal psychological, emotional, and spiritual changes that are required to maintain abstinence. The last step in AA is described as a spiritual awakening [16]. Relatedly, S/R interventions have been shown to be more efficacious than non‐S/R interventions for individuals with substance use disorders, according to a meta‐analysis conducted by Hai et al. [17]. However, the mechanism of recovery in AA remains unclear. While some participants believe that the program's spiritual foundation is essential [18], others do not [19, 20]. Analyses of the behavioral change mechanisms through which AA promotes recovery reveal that people with severe alcohol use disorder may experience a spiritual awakening [21]. However, the importance of spirituality in individuals with methamphetamine use disorder remains unknown.

The importance of spirituality may vary depending on the local culture and one's ethnicity. Although AA began as an organization comprising only White males, it has since drawn diverse memberships, including both women and men from various racial and ethnic backgrounds [22, 23, 24]. Spirituality appears to be particularly advantageous for minority groups, including Latino, African American, and American Indian populations [25]. Furthermore, religion is recognized as an important element for experiencing factors of personal recovery from mental illness [26]. In Japan, while AA and Narcotics Anonymous (NA) groups exist, Christian concepts of spiritual awakening and the role of a higher power do not align with Japanese perspectives on recovery from alcoholism [26]. It is in this context that Danshu‐kai, a self‐help organization adapted to Japanese culture, developed. In spite of being influenced by AA's 12 steps, Danshu‐kai does not make any reference to God or a higher power in the process of recovery from addiction [27]. Additionally, many Japanese individuals believe that they are nonreligious, despite engaging in a variety of religious activities [28]. In summary, the role of spirituality in recovery from addiction among Japanese individuals is unknown.

Although long‐term abstinence is not considered a primary goal for recovery from addiction, it is a need for some drug users and their families. In general, the purpose of AA is to stay sober and help others suffering from alcohol use to achieve sobriety [29]. Interviews with drug users about recovery from addiction indicate that they are motivated to maintain abstinence [30] and their understanding of recovery is related to sobriety and its maintenance [31]. In this context, Japan continues to have a zero‐tolerance policy with the slogan “Drug Abuse is Dame Zettai” (No, Absolutely, No! to Drug Abuse) [32, 33]. Furthermore, methamphetamine use is prohibited and considered a criminal problem, worsening the stigma toward drug users [34]. Because of this stigma, those who have used drugs and are suffering or trying to quit are often advised to stop using drugs by the police, employers, family, and so on [35, 36]. Taking this into account, the effect of NA on long‐term abstinence from methamphetamine through a spiritual awakening is worth investigating.

Specifically, there is a knowledge gap regarding the importance of spirituality in maintaining abstinence among individuals with methamphetamine use disorder participating in a 12‐step‐based program. Moreover, while cultural differences are a factor known to affect spirituality, to the best of our knowledge, no research has been conducted on the relationship between spirituality and abstinence in Japan. Therefore, we investigated the relationship between spirituality and abstinence from methamphetamine among Japanese individuals with methamphetamine use disorder who participated in a 12‐step‐based group.

## 2 Methods

### 2.1 Data Sources

This study used secondary data from a self‐published study (“Follow‐up Study of DARC [Drug Addiction Rehabilitation Center] Users in Japan”) [37, 38, 39]. In Japan, DARCs are private facilities that follow a 12‐step program and are primarily managed by individuals who have successfully recovered from substance use disorder. These centers function as transitional facilities and provide a supportive living environment for individuals in social recovery [40]. The data include eight follow‐up surveys from October 2016 to November 2022. The first four follow‐ups were conducted every half month from October 2016 to October 2018. The remaining four follow‐ups were conducted every 8 months because of the COVID‐19 pandemic. All DARC facilities in Japan were contacted to obtain consent from staff and facility directors to participate in this study. The patients were given an explanation of the study after providing written informed consent. They were then asked to complete a self‐report questionnaire to collect background data as a baseline survey. Face‐to‐face or telephonic interviews were used to conduct the eight follow‐up surveys.

### 2.2 Participant and Sample Selection

Two hundred ninety‐nine participants completed all eight follow‐ups over 5 years in the data from 308 participants with methamphetamine use disorder as a primary addiction in the original study. The 120 patients who successfully completed all the questionnaires for the analyses were included in this study.

### 2.3 Variables

The background characteristics of the patient population were assessed using baseline survey data collected using self‐administered questionnaires. To profile the target population, data on age, gender, dependence severity, 12‐step‐based program attendance frequency, and the presence of a sponsor were obtained. Gender, age, DAST‐20 score, spirituality, change in the frequency of NA meeting attendance between the seventh and eighth follow‐ups, NA involvement, and hospital treatment were used as the explanatory variables in the analysis. These variables were selected based on previous studies and the present sample size.

For the binary logistic regression analysis, complete abstinence from methamphetamine during the observation period was used as the response variable. If a patient used methamphetamine at any point during the follow‐up period, they were classified as nonabstinent.

Spirituality was measured using the Spiritual Rating Scale‐A (SRS‐A), which was developed for the Japanese context by Higa [41] and has demonstrated adequate reliability and validity. The SRS‐A had an alpha coefficient value of 0.82 and a reliability coefficient value of 0.72 in the original study. It measures five spirituality‐related factors, including self‐awareness and values, through 15 questions on a 6‐point Likert scale, which is less burdensome for participants to complete. The highest possible score is 75, and SRS‐A scores were used as the explanatory variable in this study.

The severity of drug‐related issues was measured using the Japanese version of the Drug Abuse Screening Test‐20 (DAST‐20), which categorizes drug dependence as nonsevere and low (scores of 0–5), intermediate (6–10), and severe (≥ 11) [42]. This variable was measured on a numerical scale ranging from 0 to 20.

The change in the frequency of NA meeting attendance was determined by a questionnaire asking how often participants participated in NA meetings in the past 6 months during the last follow‐up period. The response used a 6‐point Likert scale (1 = every day, 2 = a few times a week, 3 = once a week, 4 = once a month, 5 = almost none, and 6 = unsure). In this study, if a patient participated in an NA meeting once a week at the seventh follow‐up and a few times a week at the eighth follow‐up, the change in the frequency of NA meeting attendance was 1, indicating that the patient participated in NA meetings more often. If this number was negative, NA meetings were attended less often.

Hospital treatment was assessed using a binary question asking if a participant was currently under special treatment for substance use disorder at the time of the last follow‐up. Participants' involvement in NA was determined by NA meeting attendance, having or being a sponsor, and reading the 12‐step literature. If a participant engaged in all activities, the score was 3. If no activity was engaged in, the score was 0.

## 3 Results

A total of 120 patients who completed all surveys over 5 years were included in the analysis. Their characteristics are presented in Table 1. In addition, spirituality was analyzed by age group and gender (Table 2). Although there was only one person in the female 20–29 age group, they scored the highest (56 points). In contrast, in the male groups, the 60–69 age group had the highest mean score (49.7 points). Binary logistic regression was used to investigate the relationship between gender, age, spirituality, change in NA meeting attendance, and addiction treatment as the predictor variables and abstinence from methamphetamine as the criterion variable. The first model investigated whether there was an interaction effect between spirituality and severity of methamphetamine use disorder on complete abstinence. Neither variable had a significant interaction effect with abstinence (AOR = 1.00, 95% CI = 0.99–1.00). Subsequently, these two variables were included separately in the model. The model had a good fit (Hosmer–Lemeshow test = 10.7, df = 8, *p* = 0.22). With all other predictor variables constant, the odds of abstinence from amphetamine for 5 years increased by 1.05 (95% CI = 1.00–1.09) with a 1‐point increase in the spirituality score, and the same odds increased by 2.28 (95% CI = 1.37–3.79) for a 1‐point increase in NA involvement (Table 3). The model had an AUC value of 0.73 (*p* ≤ 0.01, 95% CI = 0.63–0.82) and was deemed a good fit.

**TABLE 1:** Characteristics of the patient population in the baseline survey.

	*N* = 120
Age (years), mean ± SD	43.2 ± 9.7
Biological sex	
Male	108 (90.0%)
Female	12 (10.0%)
Severity: DAST‐20 score, mean ± SD	13.8 ± 3.6
SRS‐A: mean ± SD	46.9 ± 10.2
Change in group attendance frequency: mean ± SD	2.8 ± 1.4
Group involvement: mean ± SD	1.8 ± 0.9
Medical treatment	
Yes	84 (70.0%)
No	36 (30.0%)

*Note:* Data are presented as *n* (%) unless otherwise indicated.

Abbreviations: DAST‐20, drug abuse screening test 20; SRS‐A, spirituality rating scale A.

**TABLE 2:** Spirituality by age group and gender.

	Male (*N*)	Female (*N*)	Total
Age			
20–29	42.0 ± 4.08 (4)	56.0 ± 0.00 (1)	44.8 ± 7.19 (5)
30–39	46.7 ± 11.41 (32)	45.89 ± 10.42 (9)	46.5 ± 11.07 (41)
40–49	47.1 ± 9.23 (41)	45.00 ± 2.83 (2)	47.0 ± 9.03 (43)
50–59	46.8 ± 12.33 (24)	—	46.8 ± 12.33 (24)
60–69	49.7 ± 6.25 (6)	—	49.7 ± 6.25 (6)
70–79	47.0 ± 0.00 (1)	—	47.0 ± 0.00 (1)

**TABLE 3:** Results of the multivariable binary logistic regression.

Variable	AOR (95% CI)	*p*
Gender (vs. male)	0.49 (0.12–1.96)	0.31
Age	1.03 (0.98–1.08)	0.24
DAST‐20	0.96 (0.86–1.08)	0.49
SRS‐A	1.05 (1.00–1.09)	0.04*
Change in group attendance	1.06 (0.68–1.65)	0.79
Group involvement	2.28 (1.37–3.79)	< 0.01*
Medical treatment (vs. none)	0.95 (0.38–2.37)	0.92
constant	0.03	

*Note:* AUC = 0.73 (*p* ≤ 0.01, 95% CI = 0.63–0.82). Variable: The response variable (abstinence) = 1.

Abbreviations: AOR, adjusted odds ratio; CI, confidence interval; DAST‐20, drug abuse screening test; SRS‐A, spirituality rating scale A.

* *p* ≤ 0.05.

## 4 Discussion

In this study, we conducted a binary logistic regression on the data of Japanese individuals in drug addiction rehabilitation centers and participated in a 12‐step‐based program for methamphetamine abuse to investigate the long‐term relationship between spirituality and drug abstinence. The results demonstrated a significant relationship between spirituality and long‐term methamphetamine abstinence. In addition, greater program involvement was significantly related to abstinence.

Consistent with a previous study on the relationship between spirituality and methamphetamine use [43], the present results confirm the importance of spirituality in recovering from methamphetamine use disorder. In Galanter et al.'s factor analysis, spirituality accounted for 29.2% of the variance, whereas social resources derived from NA support and support from other institutions accounted for 29.6% and 11.8% of the variance, respectively [43]. Notably, intoxicated individuals can behave in ways that are inconsistent with their own moral codes and values, leading to suicidal tendencies and self‐criticism; for such individuals, spirituality may facilitate self‐forgiveness toward their own maladaptive behaviors [44]. This might be one reason why the present results show that 12‐step‐based programs work on methamphetamine use disorder in the same way as for alcohol use disorder.

Interestingly, spirituality was associated with long‐term abstinence from methamphetamine in our sample. However, Japanese individuals are thought to have a poor understanding of religiosity. Previous studies report that cultural differences play a role in spirituality and that spiritual awareness tends to be lower in Japan [25, 28]. Nevertheless, Japanese individuals engage with several religions, including Buddhism, Shintoism, and Christianity. Consequently, they avoid mentioning whether they are monotheists or polytheists. Moreover, owing to the terrorist attacks in the 1990s perpetrated by the members of Aum Shinrikyo, a new religious movement founded in 1987, the word “religion” now has dangerous connotations in Japan [45]. Thus, Japanese individuals prefer to say they are nonreligious [28]. However, they also claim to have connections with deities and higher powers because of culturally ingrained beliefs that have become a normal part of their lives. For example, Danshu‐kai, a self‐help organization for alcohol use disorder, was originally developed because the spiritual aspect of the 12‐step program did not suit Japanese individuals; it includes Buddhism‐related activities, although participants do not perceive them as religious activities [28, 46]. This may explain why our results indicate a relationship between spirituality and substance use among Japanese individuals.

Furthermore, our results support the importance of having and becoming a sponsor, which is consistent with previous studies. Becoming a sponsor is believed to increase one's self‐awareness, psychological well‐being, positive social approval, status, and social competence [47]. Similarly, sponsors provide support and encouragement during personal difficulties [48]. For example, a previous study showed that having a sponsor predicted abstinence from drug and alcohol [49]. Similarly, we found that program involvement, such as sponsorship, was associated with methamphetamine abstinence.

This study had some limitations. First, the questionnaire used to measure spirituality (the SRS‐A) was designed for Japanese individuals without a high awareness of religion, and it differs from the assessment tools used in previous studies. It does not include religious aspects of spirituality to better reflect Japanese spirituality [41]. Previous studies investigating how spirituality affects alcohol abstinence asked participants whether they had had spiritual experiences [21, 50]. Although spirituality is an important factor discouraging methamphetamine use among Japanese individuals, it may differ from the spirituality of individuals from Western countries, who connect to a greater degree with the spirituality of a 12‐step program. However, the measurement we used in this study shares common factors with other scales with spirituality‐related factors, such as the Spirituality Assessment Scale, Spiritual Orientation Inventory, and Spiritual Well‐Being Scale [41, 51, 52, 53]. Thus, the SRS‐A is not unique among spirituality scales, and this finding can be applicable to other spiritual studies.

Second, the study participants belonged to a limited demographic group. They included only those who joined a DARC and practiced a 12‐step program. For example, individuals who felt that a 12‐step program did not suit them might have decided to leave the DARC, thereby not answering the questionnaires for 5 years. Because of the nature of the data used, our findings do not reveal the importance of spirituality for Japanese individuals who do not need DARCs and 12‐step program to abstain from methamphetamine use. Future research should explore the importance of spirituality among those who quit the 12‐step‐based program to further understand its role.

Third, unlike previous studies, the severity of substance use disorder was not significantly related with abstinence from methamphetamine, which can be explained by how the severity of substance use disorder was originally measured. Although the same tool (the DAST‐20) was used throughout the study, only the baseline severity of substance use disorder was measured differently compared to the other follow‐ups. The participants were asked to assign a score to the point in their life when their substance use was the most severe, not at the moment when they answered the questionnaire. Therefore, they tended to score higher at the baseline. This may explain why the severity of substance use disorder was not significantly associated with abstinence as well as why no interaction effect was observed between spirituality and severity of drug use on abstinence.

Last, a limitation of this study is its inability to account for the impact of the COVID‐19 pandemic on the relationship between spirituality and methamphetamine abstinence. The final four follow‐up assessments were conducted online during the pandemic, a period when face‐to‐face NA meetings were largely suspended and replaced by online alternatives. While the potential for differential engagement or benefit from online versus in‐person meetings exists, and the pandemic may have influenced spirituality, these factors could not be investigated due to the absence of spirituality measures prior to the final follow‐up. Thus, although the pandemic may have influenced the observed relationship between spirituality and methamphetamine use, this study could not fully address this potential confounding variable.

Our results suggest that higher levels of spirituality and greater NA involvement are significantly associated with long‐term abstinence from methamphetamine among Japanese individuals in 12‐step‐based programs. This is the first study to report such a relationship in Japan, indicating that despite cultural differences in how it is understood, spirituality plays a crucial role in long‐term abstinence from methamphetamine. The results suggest that people from non‐Western cultures and backgrounds may potentially benefit from 12‐step‐based programs because of a strengthened spirituality.

## Author Contributions

Takuya Shimane designed the preliminary experiments. Takuya Shimane and Satoshi Inoura recruited the patients and gathered the data. In addition, Takuya Shimane created a database of the participants. Takuma Ofuchi designed the study, conducted the statistical analyses, and drafted the manuscript. Takuya Shimane and Toshihiko Matsumoto reviewed the study design, statistical analyses, and manuscript development. Takuma Ofuchi prepared the initial draft of the manuscript. All authors participated in revising and finalizing the manuscript and reviewed and approved the final version for publication.

## Ethics Statement

The study protocol was reviewed and approved by the Ethics Committee of the National Center of Neurology and Psychiatry of Japan (A2016—022). Written informed consent was obtained from all participants. The study conformed to the provisions of the Declaration of Helsinki.

## Consent

Patients at rehabilitation centers were informed about the purpose of the study, and those who expressed interest were invited to participate after providing written informed consent. All participants in the original study, including those included in the present study, provided written consent. A copy of the consent form is available for review upon request.

## Conflicts of Interest

The authors declare no conflicts of interest.

## Data Availability

This study's dataset cannot be made publicly available, because data‐sharing approval has not been obtained from the Ethics Committee of the National Center of Neurology and Psychiatry. However, while raw data are not available, all the survey questionnaires, descriptive statistics for all the variables, cross‐tabulation results, and estimation‐related results are publicly accessible on the corresponding author's institutional website. Also, the data that support the findings of this study are available from the corresponding author upon reasonable request.
